# Serial crystallography is just getting started

**DOI:** 10.1063/4.0000942

**Published:** 2025-10-27

**Authors:** Aaron S Brewster, Daniel W Paley, David W Mittan-Moreau, Nicholas K Sauter

**Affiliations:** Lawrence Berkeley National Laboratory, Berkeley, CA, USA

## Abstract

When serial crystallography at XFELs was first being developed, it was unclear how it should best be used. Early experiments included studying single crystals, or weak or small crystals, or sample delivery methods that over used crystal material or under used the pulse rate. It took years of refinements to find the strengths of this method, including pump/probe experiments, room-temperature structure determination, damage-free chemistry/function measurements, exploration of dynamics, and more recently, small molecule serial femtosecond crystallography (smSFX). Now, interest in the field is exploding, with serial beamlines being constructed at synchrotrons, either from scratch or being added onto existing instruments. In this talk, several serial crystallography projects will be presented, including multi-modal methods, ligand screening at the ALS, and the transformative method of smSFX.

